# Titratable transmembrane residues and a hydrophobic plug are essential for manganese import *via* the *Bacillus anthracis* ABC transporter MntBC-A

**DOI:** 10.1016/j.jbc.2021.101087

**Published:** 2021-08-18

**Authors:** Anastasiya Kuznetsova, Gal Masrati, Elena Vigonsky, Nurit Livnat-Levanon, Jessica Rose, Moti Grupper, Adan Baloum, Janet G. Yang, Douglas C. Rees, Nir Ben-Tal, Oded Lewinson

**Affiliations:** 1 Department of Molecular Microbiology and the Rappaport Institute for Medical Sciences, Faculty of Medicine, The Technion-Israel Institute of Technology, Haifa, Israel; 2 Department of Biochemistry and Molecular Biology, George S. Wise Faculty of Life Sciences, Tel Aviv University, Tel Aviv, Israel; 3 Infectious Disease Unit, Rambam Health Care Campus, Haifa, Israel; 4 Department of Chemistry, University of San Francisco, San Francisco, California, USA; 5 Division of Chemistry and Chemical Engineering, California Institute of Technology, Pasadena, California, USA

**Keywords:** ABC transporter, virulence, transition metals, metal binding, membrane proteins, ATPase, DHD, Asp 47, His 51, and Asp 94, ICP-MS, inductively coupled plasma MS, MSA, multiple sequence alignment, NBDs, nucleotide-binding domains, SBP, substrate-binding protein, TM, transmembrane, TMDs, transmembrane domains

## Abstract

All extant life forms require trace transition metals (*e.g.*, Fe^2/3+^, Cu^1/2+^, and Mn^2+^) to survive. However, as these are environmentally scarce, organisms have evolved sophisticated metal uptake machineries. In bacteria, high-affinity import of transition metals is predominantly mediated by ABC transporters. During bacterial infection, sequestration of metal by the host further limits the availability of these ions, and accordingly, bacterial ABC transporters (importers) of metals are key virulence determinants. However, the structure–function relationships of these metal transporters have not been fully elucidated. Here, we used metal-sensitivity assays, advanced structural modeling, and enzymatic assays to study the ABC transporter MntBC-A, a virulence determinant of the bacterial human pathogen *Bacillus anthracis*. We find that despite its broad metal-recognition profile, MntBC-A imports only manganese, whereas zinc can function as a high-affinity inhibitor of MntBC-A. Computational analysis shows that the transmembrane metal permeation pathway is lined with six titratable residues that can coordinate the positively charged metal, and mutagenesis studies show that they are essential for manganese transport. Modeling suggests that access to these titratable residues is blocked by a ladder of hydrophobic residues, and ATP-driven conformational changes open and close this hydrophobic seal to permit metal binding and release. The conservation of this arrangement of titratable and hydrophobic residues among ABC transporters of transition metals suggests a common mechanism. These findings advance our understanding of transmembrane metal recognition and permeation and may aid the design and development of novel antibacterial agents.

Owing to the limited chemical reactivity of amino acid side chains, metals such as Cu^1/2+^, Zn^2+^, Mn^2+^, and Fe^2/3+^ are indispensable cofactors for the structure and function of many enzymes and structural proteins, and it is estimated that metalloproteins comprise 30 to 40% of most proteomes (1, 2). However, metal ions are also toxic, and therefore, all organisms maintain a strict balance between essential uptake and avoiding toxic overload (3). This necessity places trace metals at the crossroads of host–pathogen interactions: to fight infections, hosts use both metal deprivation and overload in a tissue-specific manner (4, 5, 6). In turn, to maintain and control their intracellular metal quota, bacterial pathogens evolved elaborate uptake and extrusion systems (7, 8, 9).

For the high-affinity acquisition of essential trace metals, bacteria primarily utilize ABC transporters, and it is therefore not surprising that a large number of ABC transporters (importers) of trace metals have been identified as key bacterial virulence determinants (10, 11, 12, 13, 14, 15, 16). ABC transporters comprise one of the largest superfamilies of proteins of any proteome (17, 18). They utilize ATP hydrolysis to transport molecules across biological membranes and are present in all extant phyla with 79 genes in *Escherichia coli*, ∼120 in higher plants, and 48 in humans (19). The transported molecules are extremely diverse, ranging from small molecules (such as metal ions, sugars, and amino acids) to large and bulky compounds (peptides, proteins, organometal complexes, and antibiotics).

ABC transporters consist minimally of four domains: two transmembrane domains (TMDs) and two cytoplasmic nucleotide-binding domains (NBDs) (20). Binding and hydrolysis of ATP at the NBDs power the translocation of substrates through the permeation pathway formed by the TMDs. A given ABC transporter may function as either an exporter or an importer. ABC transporters that function as importers depend on a high-affinity substrate-binding protein (SBP) that delivers the substrate to the cognate transporter (21, 22, 23).

The *mntBCA* operon of the potentially lethal human pathogen *Bacillus anthracis* encodes an ABC import system which has been shown to be an essential virulence determinant: deletion of MntA (hereafter baMntA), the system’s SBP, yields a completely nonvirulent strain, less virulent than the Sterne strain used for vaccination (10). *In vivo* analysis suggested that baMntBC-A imports manganese and/or iron (10), whereas *in vitro* studies demonstrated that baMntA does not bind iron but rather binds a variety of other metals, leaving the recognition specificity of the system unclear (24). The mechanism of action of baMntBC-A (or of any other ABC transporter of transition metals) is unknown, and this paucity of information contrasts with their clinical relevance (13, 14, 16, 25, 26, 27, 28).

Here, we present a structure–function analysis of the *B. anthracis* transition metal ABC transporter baMntBC-A. Using a combination of experimental and computational approaches, we determined its transport specificity, identified essential transmembrane (TM) residues that potentially form a TM metal recognition site, and provide the first description of its ATP hydrolysis cycle.

## Results

### Transport specificity of baMntBC-A

In a previous study, we determined the metal-binding spectrum of baMntA of *B. anthracis* (24), the cognate SBP of the baMntBC transporter. We found that baMntA binds several transition metals with affinities ranging from 10^−6^ to 10^−8^ M. The highest affinity was toward Co^2+^ (*K*_*D*_ ∼ 5 × 10^−8^ M) and the lowest toward Ni^2+^ (*K*_*D*_ ∼ 4 × 10^−6^ M), and intermediate binding affinities (*K*_*D*_ 3–5 × 10^−7^ M) were measured for Mn^2+^, Zn^2+^, and Cd^2+^.

Although substrate binding by the SBP is essential for transport (22, 23, 29, 30), it is not sufficient: several studies have now established that the SBP may bind ligands that are not transported by the transporter (31, 32, 33).

To investigate the transport specificity of baMntBC-A, we cloned the coding region of the *B. anthracis mntBCA* transporter and inserted it into an IPTG-inducible *Bacillus subtilis* chromosomal integration vector (34).

**Figure 1:**
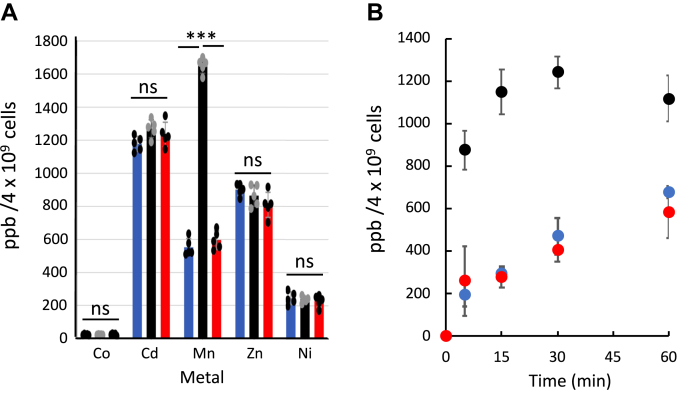
**baMntBC-A imports only manganese.***A*, midexponential phase cultures of *Bacillus subtilis* cells transformed with either a control plasmid (*blue bars*) or a plasmid encoding WT (*black bars*) or E163A (*red bars*) baMntBC-A were incubated for 15 min with 50 μM of the sulfate salts of the indicated metals. Cells were then harvested and washed with PBS-EDTA buffer, and their intracellular metal content was determined by ICP-MS. Data are the mean ∓ standard deviation of the mean of replicates (n = 5). Normal distribution of the data was verified by the Shapiro–Wilk test (α = 0.05), and statistics were calculated using one-way ANOVA. ∗∗∗*p* < 0.001. *B*, shown is the time-dependent accumulation of manganese (added at 50 μM) in control cells (*blue circles*) or in cells expressing WT (*black circles*) or E163A (*red circles*) baMntBC-A. ICP-MS, inductively coupled plasma MS; ns, not significant.

We then transformed WT *B. subtilis* with either the empty chromosomal-integration plasmid or the same plasmid harboring the baMntBC-A transporter. Various metals were added to midexponential phase cultures, and intracellular metal content was then measured by inductively coupled plasma MS (ICP-MS). We first tested the accumulation of Co^2+^ (added as CoSO_4_) because it is the metal that is bound with the highest affinity by baMntA (24), the SBP of the system. We found that the intracellular concentration of Co^2+^ was independent of the expression of baMntBC-A. Similarly, the intracellular accumulation of Ni^2+^, Zn^2+^, and Cd^2+^ was also unchanged upon expression of baMntBC-A (Fig. 1*A*). In contrast, when MnSO_4_ was added to the growth media, the intracellular accumulation of Mn^2+^ in cells expressing baMntBC-A was ∼2.5-fold higher than in control cells (Fig. 1*A*). Dose-dependent experiments revealed that this fold difference was maintained over a relatively broad range (1–250 μM) of manganese concentrations (Fig. S1).

These results suggest that despite the broad metal-recognition spectrum of the SBP (MntA) baMntBC-A seems to transport (import) only Mn^2+^.

To confirm that the increased accumulation of manganese is specific to the transport (import) activity of baMntBC-A, we repeated the experiments with a baMntBC-A variant that carries a mutation in the glutamate of the Walker B motif (E163A). In all ABC transporters, this conserved glutamate serves as the catalytic base for cleavage of the γ-phosphate bond and is therefore absolutely essential for ATP hydrolysis and transport (20, 35, 36, 37, 38). As shown, despite WT-like expression levels of this mutant, the intracellular manganese content of cells expressing baMntBC-A (E163A) was identical to that of cells transformed with the control plasmid (Fig. 1*A* and Fig. S2*A*). These results suggest that the increased accumulation of manganese in cells expressing WT baMntBC-A is indeed due to the ATPase-dependent transport (import) activity of the transporter.

Time-dependent metal uptake assays revealed that the transport is quite rapid, and significant baMntBC-A-mediated accumulation of manganese was observed in the expressing cells already after 5 min of activity (Fig. 1*B*).

**Figure 2:**
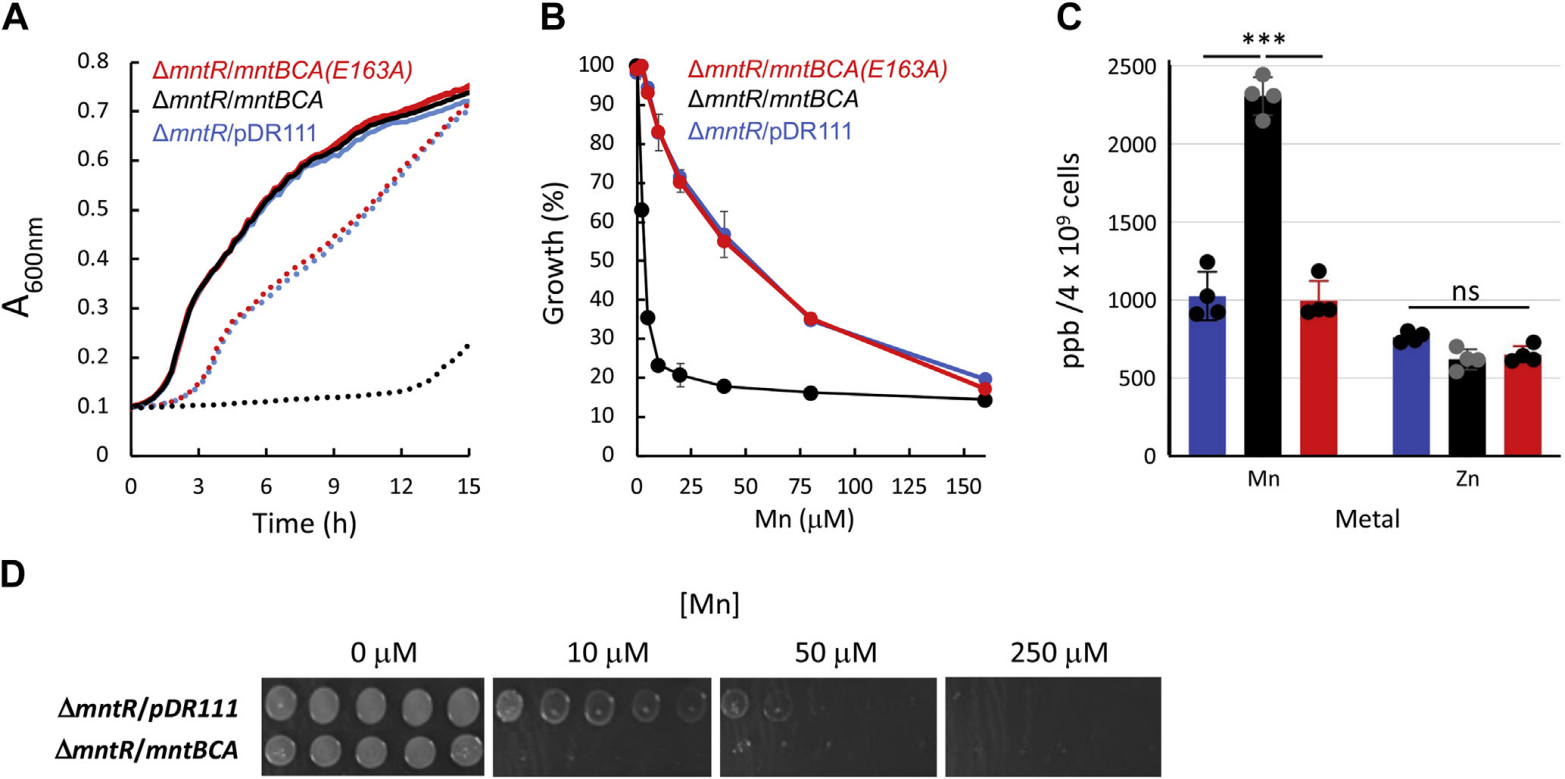
**Expression of baMntBC-A leads to increased manganese sensitivity.***A*, cultures of *ΔmntR* cells transformed with the pDR111 control plasmid (*blue curves*) or with the same plasmid encoding WT baMntBC-A (*black curves*) or the ATPase-deficient mutant baMntBC-A (E163A, *red curves*) were grown in LB media supplemented with IPTG in the absence (*solid lines*) or presence (*dotted lines*) of 5 μM MnSO_4_. Shown are representative results of experiments conducted at least three times. *B*, cells were grown for 12 h in the presence of the indicated concentration of MnSO_4_ and their final absorbance is expressed as percentage growth relative to the growth observed in the absence of MnSO_4_. Shown are the averages of biological triplicates, and the error bars (shown unless smaller than icons) represent the SDs of the mean. *C*, midexponential phase cultures of *ΔmntR* cells transformed with either a control plasmid (*blue bars*) or a plasmid encoding WT (*black bars*) or E163A (*red bars*) baMntBC-A were incubated for 15 min with 50 μM of either MnSO_4_ or ZnSO_4_ as indicated. Cells were then harvested and washed with PBS-EDTA buffer, and their intracellular metal content was determined by ICP-MS. Data are the mean ∓ standard deviation of the mean of replicates (n = 4). Normal distribution of the data was verified by the Shapiro–Wilk test (α = 0.05), and statistics were calculated using one-way ANOVA. ∗∗∗*p* < 0.001. *D*, *ΔmntR* cells were transformed with a pDR111 control plasmid or with the same plasmid harboring the complete baMntBC-A operon, as indicated. Cultures were grown to the midexponential phase and then diluted to *A*_600_ of 0.01. Drops of 2.5 μl were spotted in serial 10-fold dilutions from left to right onto LB-agar plates supplemented with IPTG in the presence of the indicated concentration of MnSO_4_. Shown are representative results of experiments conducted at least three times. The *dashed line* represents a crop from a single unprocessed image. ICP-MS, inductively coupled plasma MS; ns, not significant.

To complement the Mn^2+^ transport assays described above, we performed metal-sensitivity growth assays using the previously described manganese-sensitive *ΔmntR B. subtilis* strain (8, 39). We reasoned that if indeed baMntBC-A functions as a manganese importer its activity will be readily detected in this manganese-sensitive strain. We therefore transformed *ΔmntR* cells with either an empty chromosomal-integration plasmid (pDR111) or with the same plasmid that contains the complete baMntBC-A operon under control of the *lac* promoter. As shown in Figure 2*A*, in the absence of exogenously added manganese, the growth of *ΔmntR B. subtilis* was not affected by the expression of baMntBC-A, suggesting that the plasmid-driven expression of baMntBC-A is in itself not inhibitory to cell growth. However, even if low concentrations of manganese were added (5 μM MnSO_4_), cells that expressed WT baMntBC-A showed a dramatic growth attenuation (Fig. 2*A*). Importantly, cells expressing the inactive variant (E163A) did not show increased manganese sensitivity. This baMntBC-A-conferred manganese hypersensitivity was observed over a broad range of concentrations (2–160 μM, Fig. 2*B*). In line with their increased manganese sensitivity, *ΔmntR* cells expressing WT baMntBC-A accumulated more manganese than control cells or baMntBC-A (E163A)-expressing cells, whereas their intracellular zinc concentration remained unchanged (Fig. 2*C*).

To complement these studies that were conducted in liquid media, we performed drop-dilution tests in solid media. In line with the results obtained in liquid media, expression of baMntBC-A in the *ΔmntR* strain led to increased manganese sensitivity (Fig. 2*D*).

In a previous study (24), we identified three residues that comprise the metal-binding site of baMntA: mutations in either H141 or E207 completely abolished manganese recognition, whereas a mutation in H69 led to ∼10-fold reduction in binding affinity. To further substantiate the link between the observed manganese-sensitivity phenotype and expression of functional baMntBC-A, we tested the manganese sensitivity of mutants H141A, E207A, and H69A.

**Figure 3:**
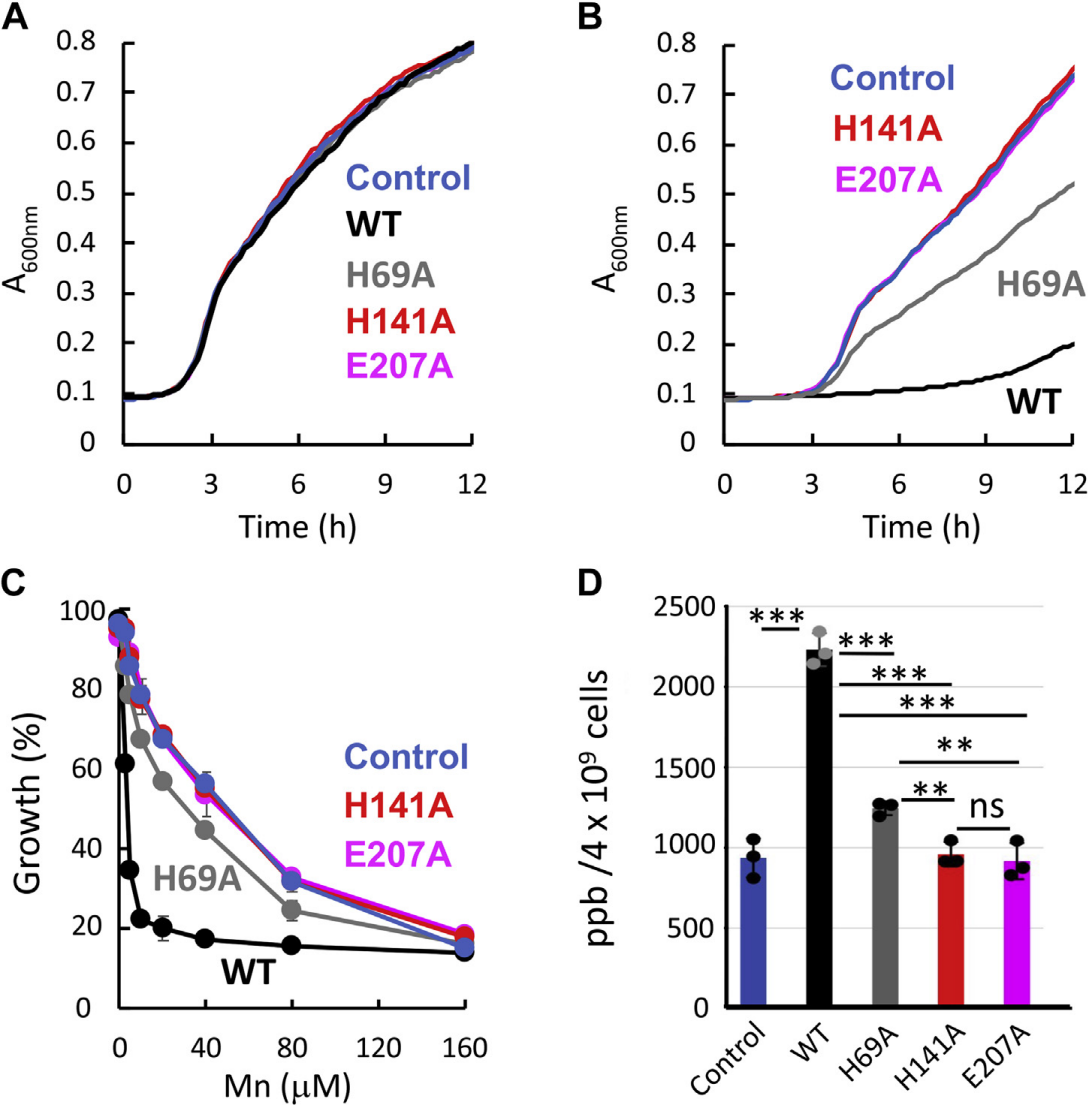
**Mutations of the metal-binding residues of baMntA abolish the manganese sensitivity.** Cultures of *ΔmntR* cells transformed with the pDR111 control plasmid or with the same plasmid encoding WT or mutant baMntBC-A (as color indicated) were grown in LB media supplemented with IPTG in the absence (*A*) or presence (*B*) of 5 μM MnSO_4_. Shown are representative results of experiments conducted at least three times. *C*, *ΔmntR* cells were grown for 12 h in the presence of the indicated concentration of MnSO_4_, and their final absorbance is expressed as percentage growth relative to the growth observed in the absence of MnSO_4_. Shown are the averages of biological triplicates, and the error bars (shown unless smaller than icons) represent the SDs of the mean. *D*, midexponential phase cultures of *Bacillus subtilis* cells transformed with either a control plasmid or the same plasmid encoding WT or mutant baMntBC-A (as color indicated) were incubated for 15 min with 50 μM of MnSO_4_. Cells were then harvested and washed with PBS-EDTA buffer, and their intracellular metal content was determined by ICP-MS. Data are the mean ∓ standard deviation of the mean of replicates (n = 3). Normal distribution of the data was verified by the Shapiro–Wilk test (α = 0.05), and statistics were calculated using one-way ANOVA. ∗∗∗*p* < 0.001; ∗∗*p* < 0.05. ICP-MS, inductively coupled plasma MS; ns, not significant.

In the absence of manganese, the growth of *ΔmntR* cells expressing these mutants was indiscernible from that of cells expressing WT baMntBC-A or from that of cells transformed with the control plasmid (Fig. 3*A*). In line with their complete inability to bind manganese (24), the manganese sensitivity of the fully inactive mutants (H141A and E207A) was identical to that of the cells that did not express baMntBC-A (Fig. 3, *B* and *C*), and, as expected, these cells did not overaccumulate manganese (Fig. 3*D*). This lack of activity of mutants H141A and E207A could not be attributed to changes in expression levels caused by the point mutations (Fig. S2*A*). Notably, in line with its *in vitro* ∼10-fold reduced manganese-binding affinity, expression of mutant H69A conferred modest manganese sensitivity, which was significantly different from that of the two fully inactive mutants and from that of the WT protein. ICP-MS analysis revealed that the intracellular manganese concentration of cells expressing mutants H141A and E207A was comparable with that of cells that did not express baMntBC-A, whereas the intracellular manganese concentration found in cells expressing mutant H69A was slightly higher (Fig. 3*D*).

Taken together, the results with the four mutations that compromise either the ATPase activity of baMntBC (Fig. 2) or manganese binding by baMntA (Fig. 3) support the conclusion that the manganese hypersensitivity observed in cells that express WT baMntBC-A is indeed due to active import of manganese by baMntBC-A.

### Zn^2+^, but not other metals, inhibits transport of Mn^2+^

**Figure 4:**
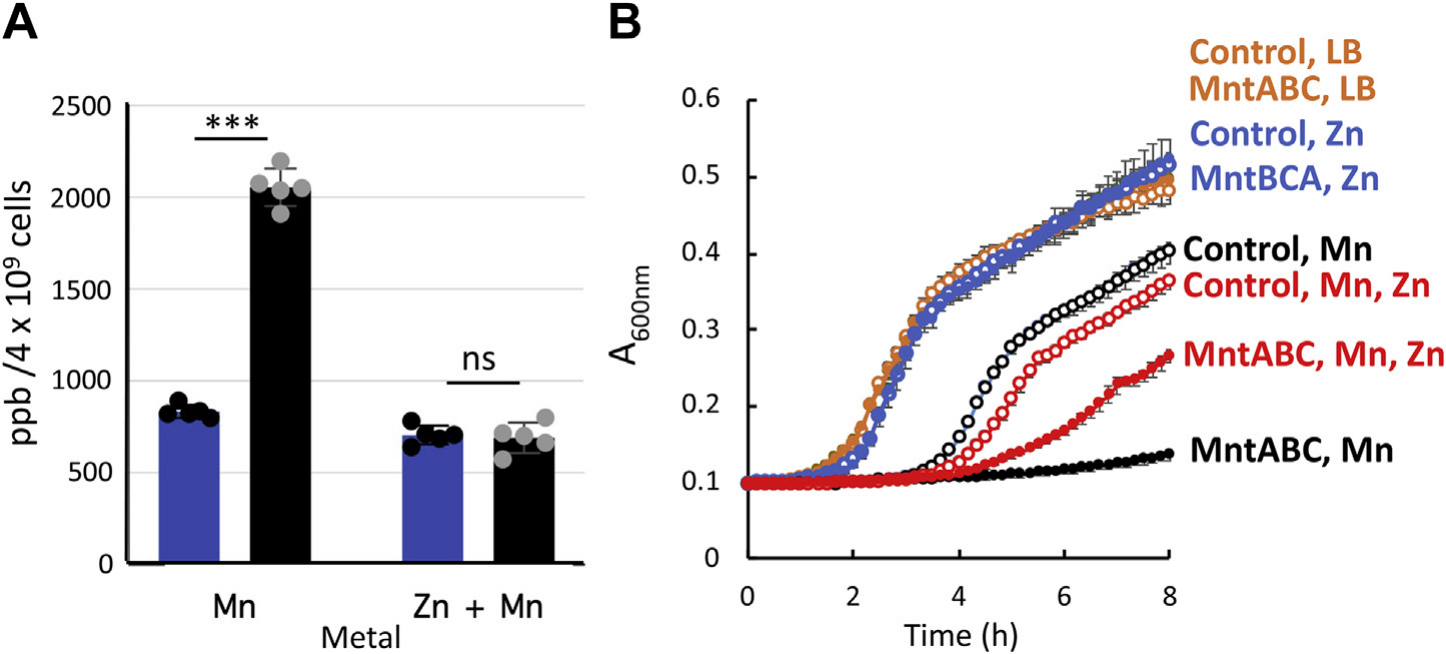
**Zinc inhibits baMntBC-A-mediated transport of manganese.***A*, midexponential-phase cultures of WT *Bacillus subtilis* 168 cells transformed with either a control plasmid (*blue bars*) or a plasmid harboring the complete baMntBC-A operon (*black bars*) were incubated for 15 min with 100 μM of the indicated metals. Cells were then harvested and washed with PBS-EDTA buffer, and the intracellular metal content was determined by ICP-MS. Data are the mean ∓ standard deviation of the mean of replicates (n = 5). Normal distribution of the data was verified by the Shapiro–Wilk test (α = 0.05), and statistics were calculated using one-way ANOVA. ∗∗∗*p* < 0.001. *B*, cultures of *Bacillus subtilis ΔmntR* cells transformed with a control plasmid (*open symbols*) or a plasmid harboring the complete baMntBC-A operon (*full symbols*) were grown in LB media (*orange symbols*) or LB media supplemented with 10 μM MnSO_4_ (*black symbols*), 50 μM ZnSO_4_ (*blue symbols*), or their combination (*red symbols*). Shown are averages of biological triplicates, and the error bars (shown unless smaller than the symbols) represent SDs of the mean. ICP-MS, inductively coupled plasma MS; ns, not significant.

In a previous work, we observed that Mn^2+^ and Zn^2+^ compete for binding to the same metal-binding site in baMntA (24). In the same work, we also observed that the SBP–Zn^+2^ complex is very stable and that Zn^2+^ is released from the SBP at a very slow rate. Although these experiments were conducted in the absence of the transporter, this suggested that Zn^2+^ is a nontransportable metal. Indeed, as shown above (Fig. 1*A*), Mn^2+^ is transported by baMntBC-A, whereas Zn^2+^ is apparently not. Very similar findings were originally reported for PsaA, the Mn^2+^ SBP of *Streptococcus pneumoniae*, and therefore, the susceptibility of this bacterium to Zn^2+^ was attributed to the Zn^2+^-mediated inhibition of Mn^2+^ uptake (11, 40). Based on these observations, it was suggested that Zn^2+^ may be used as an inhibitor of bacterial manganese ABC importers such as baMntBC-A. To test this, we measured the intracellular accumulation of Mn^2+^ in cells that were preincubated with Zn^2+^. As shown in Figure 4*A*, preincubating baMntBC-A-expressing cells with Zn^2+^ reduced their intracellular Mn^2+^ content to levels that were similar to those observed in control cells that did not express baMntBC-A. These results suggest that Zn^2+^ inhibits the Mn^2+^ uptake activity of baMntBC-A and therefore may alleviate the manganese hypersensitivity of baMntBC-A-expressing cells.

To test this, we grew control and baMntBC-A-expressing cells in the presence of Mn^2+^, Zn^2+^, and combinations thereof. As shown, addition of 50 μM Zn^2+^ was only marginally inhibitory to growth, and this effect was identical in control and baMntBC-A-expressing cells (Fig. 4*B*, compare orange and blue traces). This is not surprising in light of the lack of Zn^2+^ import activity of baMntBC-A (Fig. 1*A*). As expected, cells expressing baMntBC-A were much more sensitive to the addition of 10 μM Mn^2+^ than control cells (Fig. 4*B*, black symbols). In cells that did not express baMntBC-A, the addition of 50 μM Zn^2+^ on top of 10 μM Mn^2+^ led to a modest (∼10%) yet statistically significant greater inhibition (Fig. 4*B*, open red symbols). In contrast, in baMntBC-A-expressing cells, the addition of 50 μM Zn^2+^ on top of 10 μM Mn^2+^ led to ∼4-fold higher growth (Fig. 4*B*, full red symbols). These results support the notion that as reported for PsaBCA of *Streptococcus pneumonia* (11, 41), Zn^2+^ inhibits the Mn^2+^ import activity of baMntBC-A. In addition to Mn^2+^ and Zn^2+^, MntA also binds Ni^2+^ and Co^2+^ (24). We therefore tested whether these metals could also serve as MntBC-A inhibitors. Surprisingly, despite its higher binding affinity (∼10-fold higher than toward either Mn^2+^ or Zn^2+^, (24)), Co^2+^ had no inhibitory effect, and similar lack of inhibition was also observed with Ni^2+^ (Fig. S3). The implications and source of the specificity of Zn^2+^ inhibition is discussed later in the article (see Conclusions).

### Model structure of baMntBC and identification of a potential TM metal-coordination site

To gain insight on the structure of baMntBC and its possible transport mechanism, we attempted to model it, which proved to be nontrivial. The baMntBC transporter is comprised of two identical subunits of MntC that form the TMDs, in complex with two identical subunits of MntB that form the NBDs. The structure of the NBDs is highly conserved among the superfamily of ABC transporters (20, 42, 43), and with query-template sequence identity close to 30%, we could use standard homology modeling to model MntB.

Unlike the NBDs, the structure and sequence of the TMDs of ABC transporters can vary significantly (42, 44). Indeed, the modeling of MntC proved to be challenging as only remote homologs are available as templates. Another problem with these templates is that they all transport substrates that are much larger than manganese, making the modeling of the translocation pathway particularly challenging. With sequence identity of only 13% to 15% to the available templates, there is uncertainty even regarding the exact number of TM helices. To overcome these challenges, we combined various computational tools to produce multiple model structures of MntC (see Experimental procedures). The result of these efforts was three models based on three different templates: (1) *Haemophilus influenza’s* putative molybdate/tungstate importer MolBC in an inward-facing conformation (PDB ID: 2NQ2; chains A and B) (45) that resulted in a semiopen MntC model; (2) *Yersinia pestis’s* heme importer HmuUV in an outward-apo conformation (PDB ID: 4G1U; chains A and B) (46) that resulted in an occluded state model; and (3) *E. coli’s* vitamin B_12_ importer in an outward-facing adenylyl imidodiphosphate-bound conformation (PDB ID: 4R9U chains A and B) (47) that produced an open conformation model. To further assess MntC’s model we also produced a template-independent model using trRosetta (48) that predicts inter-residue distance and orientation distributions based on neural networks. Both methods (trRosetta and homology modeling) converged to a similar structure with almost identical assignment of TM helices (Figs. S4 and S5). Furthermore, all models were consistent with the expected evolutionary conservation pattern, where the protein’s core is composed almost exclusively of conserved residues while its periphery is enriched with variable residues (Figs. S4 and S5). Nevertheless, owing to the low query-to-template sequence identity and the ensuing model uncertainty, in the following structural analysis, we considered only features that were observed in all three models irrespective of the template used.

**Figure 5:**
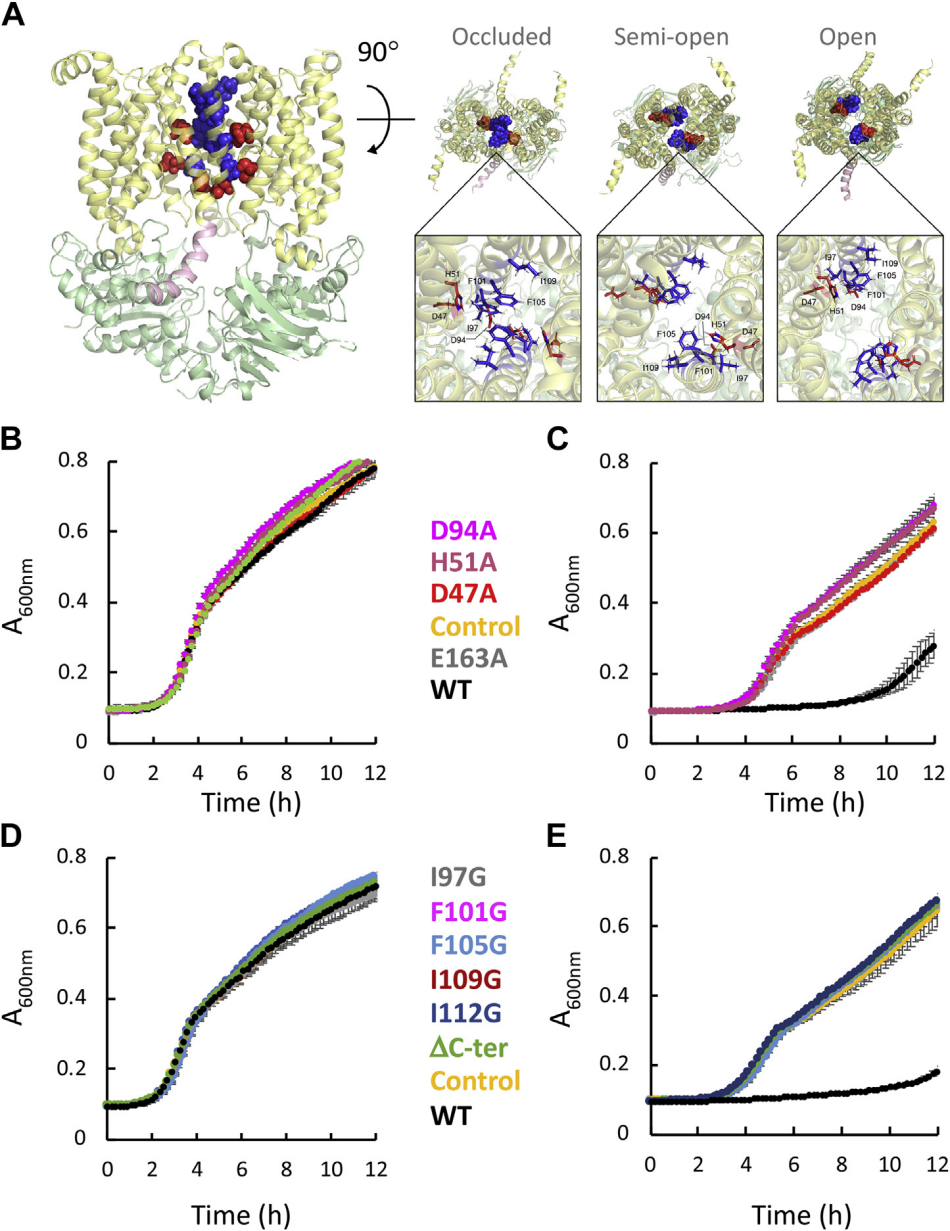
**Essential titratable residues line the translocation cavity of baMntBC.***A*, shown are *side* (*left*) and *top* (all other) views of semitransparent cartoon representations of the baMntBC models. The two images on the *left* are of the model that is based on the structure of HmuUV, depicting an occluded conformation. BtuD is colored *lime*, BtuC in *light yellow*, and the C-terminal helical extension in *salmon pink*. The two images on the *right* are based on the structures of MolBC and BtuCD and depict a semiopen and open conformations, respectively. Residues of interest are shown as either *spheres* (full-size images) or *sticks* (zoomed images). The twin charged triads, each composed of Asp 47, His 51, and Asp 94, are shown in *red* and the “hydrophobic ladder” residues (Ile97, F101, F105, Ile109, and Ile112) are in *blue*. *B*–*E*, cultures of *ΔmntR* cells were grown in the absence (*B* and *D*) or presence (*C* and *E*) of 10 μM MnSO_4_. In the absence of manganese, WT baMntBC-A and all tested mutants grew similarly. In the presence of manganese mutations in the putative metal-binding residues Asp 47, His 51, and Asp 94 (*C*) or in hydrophobic seal residues I97, F101, F105, I109, I112, or truncation of the C-terminal helix (*E*) abolished the activity of baMntBC-A and the growth of these mutants was similar to that of controls cells that did not express baMntBC-A or cells expressing the inactive mutant E163A.

The passage of a charged molecule through a membrane protein often requires coordination by membrane-embedded charged residues that neutralize the translocated charge (chapter 7 in (49)). We hypothesized that this is especially true for transport of manganese, which has one of the highest charge densities of the biologically active divalent metals. Indeed, the model shows six titratable residues that line the transmembrane translocation cavity (Fig. 5*A*, red spheres), comprised of two triads of Asp 47, His 51, and Asp 94 (DHD), where each triad is contributed by a different monomer of MntC. These three residues are highly conserved among the 430 baMntC homologs we analyzed (Fig. S6), with a score of 9 in ConSurf’s evolutionary conservation scale (1 being the most variable and 9 the most conserved, (50)). Interestingly, none of these residues is conserved in any of the templates used for the modeling and, to the best of our knowledge, in any other subgroup of ABC transporters. In addition, Asp and His residues are among the most common manganese-coordinating residues (51). Considering the preferred coordination number of 5 to 6 for manganese (51), the two DHD triads could provide a possible manganese coordination site. Of note, His and Asp residues were demonstrated to directly coordinate binding of Mn^2+^ by baMntA, the system’s SBP (24).

To test if residues DHD have a role in manganese transport, we generated mutants with alanine substitutions of these three putative metal-binding residues. Of note, because MntBC is a heterodimer of homodimers, a single substitution at the gene level results in two identical amino acid mutations at the protein level. Next, we compared the activity of these single alanine mutants with that of WT baMntBC using the Mn^2+^ sensitivity assay described above. As shown in Figure 5*B*, in the absence of manganese, the growth of control cells, cells expressing WT baMntBC, or the tested mutants was indistinguishable. In the presence of manganese (Fig. 5*C*), mutations D47A, H51A, and D94A completely abolished the baMntBC-A-mediated Mn^2+^ sensitivity, and the growth of these mutants was identical to that of the vector control and to that of the inactive E163A mutant (which carries a mutation in the essential glutamate of the Walker B motif). The lack of activity of the mutants was not due to improper expression, as their membrane-fraction expression was similar to that of the WT protein (Fig. S2*A*). These results demonstrate that the titratable residues are essential for Mn^2+^ transport and support the suggestion that they may be involved in TM metal coordination.

The model shown in Figure 5*A* is based on the structure of the heme transporter HmuUV (46), depicting baMntBC in an occluded conformation. In this conformation, hydrophobic residues (Ile97, F101, F105, Ile109, and Ile112) from TM helices 5 and 5′ form a “hydrophobic ladder” that effectively seals the metal-binding site to the extracellular side of the membrane (Fig. 5*A*, blue spheres). Interestingly, the other two models (based on apo MolBC and AMP-PNP-bound BtuCD) depict baMntBC in intermediate stages of opening toward the extracellular environment, where the width of the narrowest point in the hydrophobic seal increases from 3.6 Å (occluded) to 5.4 Å (semiopen), and finally to 11.3 Å (fully open, top views in Fig. 5*A*, as indicated). To test if the bulky residues that comprise the hydrophobic seal are important for transport, we generated single point glycine substitutions. Glycine was chosen because it is the smallest amino acid. Remarkably, replacement of any of the bulky hydrophobic seal residue with glycine completely abolished the activity of baMntBC (Fig. 5, *D* and *E*), despite normal membrane-fraction expression of the mutants (Fig. S2*B*). The relative motions of the residues that comprise the hydrophobic seal and of those that comprise the charged triads are expected to play an important role in the transport mechanism, as will be discussed later.

Of note, according to all 3D models, the TMDs of baMntBC are comprised of nine TM helices per monomer (Fig. 5*A* and Fig. S4), in contrast to the ten TM helices observed in all crystal structures of baMntBC homologs (45, 46, 47). The suggestion that the number of baMntBC TM helices differs from ten is also supported by all of the TM prediction tools we tested (Fig. S6). Furthermore, all three homology models of baMntBC, as well as the trRosetta template-free model, show an elongated C-terminal TM helix that extends well into the cytoplasm (Fig. 5*A*). Given that both the N and C termini were modeled using an *ab initio* approach (see Experimental procedures), the quality of the model in these regions is questionable. Nevertheless, the amino acid composition of the C terminus is not amphipathic (Fig. S7) and therefore not expected to lie at the membrane–cytosol interface. In comparison, the N-terminal TM helix is clearly amphipathic (Fig. S7) and as depicted in the model is expected to lie at the membrane–extracellular interface (Fig. 5*A* and Figs. S4–S7). Further support for the cytosolic location of the C-terminus helical extension is provided by the fact that out of the 21 amino acids that comprise it, six are titratable and six are polar. The net charge of this helical extension is +5, and its cytoplasmic location would be in line with the “positive inside rule” (52, 53). This charge distribution is very different from that observed in all other predicted TM helices of MntC that are heavily enriched in hydrophobic residues (Figs. S6 and S7). To test if the cytoplasmic extension of this C-terminal TM helix was important for function, we truncated it at the membrane–cytosol interface to generate the MntBC-ΔC mutant. Surprisingly, the truncation did not affect the membrane-expression of MntBC (Fig. S2*B*). However, as judged by manganese-sensitivity assays, the truncated variant was completely inactive (Fig. 5, *D* and *E*, green traces).

### Uncoupled ATPase activity of baMntBC

ABC transporters that function as importers are divided into two subgroups: type I importers typically import sugars, amino acids, and peptides, whereas type II systems import organometallic complexes such as heme, siderophores, and vitamin B_12_ (20, 42, 54, 55). These two subclasses have been shown to operate by very distinct mechanisms. One central distinctive mechanistic feature is the basal rate of ATPase activity and its modulation by the SBP.

**Figure 6:**
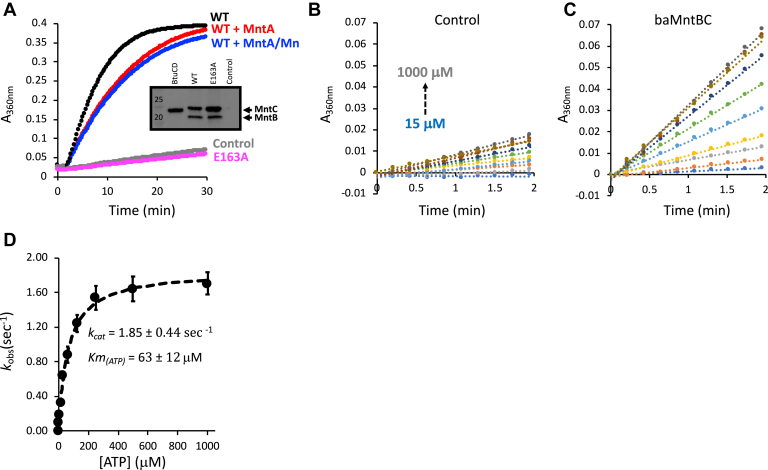
**Uncoupled ATP hydrolysis by baMntBC.***A*, 15 μg of inverted membrane vesicles were incubated for 2 min with 1 mM ATP, and to initiate hydrolysis, 2 mM MgSO_4_ was injected at 2 min. The rate of release of inorganic phosphate was determined by continuous monitoring of the 360 nm absorbance of the solution using the EnzChek kit. Shown is the activity measured for vesicles containing WT baMntBC (*black*), WT baMntBC+ baMntA (*red*), WT baMntBC+ 10 μM baMntA+ 50 μM MnSO_4_ (*blue*), mutant E163A (*magenta*), or control vesicles (*gray*). Results are representative of experiments conducted at least three times. The inset shows an immunoblot of SDS page of the vesicles, and BtuCD (used as benchmark for high expression level) and WT or mutant E163A are shown as indicated. *B* and *C*, initial rates of hydrolysis of 15 to 1000 μM ATP were measured (in the presence of baMntA and MnSO_4_) for control (*B*) and WT baMntBC (*C*) vesicles. *D*, the initial rates of ATP hydrolysis measured in panel *B* for the control vesicles were subtracted from the rates measured for WT baMntBC in panel *C*. The net values were plotted as a function of the ATP concentration, and the data were then fit using the Michaelis–Menten equation (*dashed line*). Also shown are the mean values (n = 3) of the kinetic rate constants, and the error bars represent SDs of the mean.

In type I systems, the basal rates of ATP hydrolysis are very low, and these are greatly stimulated (10- to 30-fold) upon docking of the substrate-loaded SBP (33, 56). In contrast, type-II systems have very high rates of basal ATP hydrolysis that are largely insensitive to substrate loading (46, 57, 58). The extent of substrate modulation of ATPase activity in transition metal ABC importers such as baMntBC is unknown. To study this phenomenon, we codon-optimized the nucleotide sequence of baMntBC for expression in *E. coli* and synthetically generated codon-optimized coding regions for *mntBC* (GenScript). We then prepared inverted membrane vesicles (59) from cells transformed with an empty control plasmid or plasmids encoding WT or mutant (E163A) baMntBC. We reasoned that owing to the high content of baMntBC in these vesicles (inset in Fig. 6*A*), we may be able to measure its ATPase activity without the need of detergent-mediated extraction/purification with its possible pitfalls (60). Indeed, as shown in Figure 6*A*, the ATPase activity of baMntBC-inverted membrane vesicles was ∼5-fold higher than that of vesicles prepared from control cells that were transformed with an empty control plasmid. To verify that this activity is indeed specific to baMntBC, we repeated these experiments with the ATPase-deficient mutant baMntBC (E163A). Despite the high protein content of baMntBC (E163A) vesicles (inset in Fig. 6*A*), their ATPase activity was identical to that of the control vesicles that did not contain any baMntBC (Fig. 6*A*).

We next used the vesicles system to investigate the effects of the SBP on ATP hydrolysis by the transporter. For this, codon-optimized baMntA was overexpressed and purified in *E. coli* as previously described (24) and incorporated into the vesicles’ lumen at approximately ∼10-fold molar excess over baMntBC (see Experimental procedures for details). The SBP did not stimulate the ATPase activity of the transporter, and its incorporation led to a mild inhibitory effect (Fig. 6*A*). We then repeated these experiments incorporating also manganese into the vesicles’ lumen at a 50:10:1 manganese-baMntA-baMntBC molar ratio and observed a similarly modest inhibitory effect (Fig. 6*A*). Collectively, these results suggest that similar to the type II ABC importer BtuCD, yet unlike the type I ABC importers MalFGK, YecSC, and HisPQM, baMntBC has high basal ATPase activity that is not further stimulated by the SBP–substrate complex (33, 36, 56, 57, 61).

To determine the kinetic rate constants of ATP hydrolysis, we measured the initial rates of activity under a range of ATP concentrations. As shown, at ATP concentrations of 15 to 1000 μM, the initial rates of ATP hydrolysis were linear for at least 2 min (Fig. 6, *B* and *C*). To determine the specific ATPase activity of baMntBC, we subtracted the corresponding activity measured in the control vesicles. The net rates of ATP hydrolysis were then plotted as a function of ATP concentration, and the data were fit using the Michaelis–Menten equation (Fig. 6*D*). Adding the term for the Hill coefficient yielded an n_HILL_ = 1.08, indicating that the two ATP-binding sites of baMntBC do not hydrolyze ATP cooperatively. This differs from the cooperative ATP hydrolysis that was reported for both type I and type II ABC importers (*e.g.*, MetNI, HisPQM, MalFGK, YecSC, and BtuCD) (33, 36, 61, 62, 63). Notably, even in a membrane environment, baMntBC has very high rates of uncoupled (absence of the substrate and SBP) ATPase activity (*k*_*cat*_ = 1.85 s^−1^), similar to the SBP–substrate–stimulated ATPase rates reported for type I importers (56, 61).

## Conclusions

In recent years, it is becoming increasingly clear that manganese plays an important role in host–pathogen interactions. Transport and homeostasis of manganese was shown to be essential for the virulence of key bacterial pathogens, such as *Enterococcus faecalis*, *Staphylococcus aureus*, *group A Streptococcus*, *Acinetobacter baumannii*, *Mycobacterium tuberculosis*, *S. pneumonia*, and many others (14, 15, 16, 25, 64, 65, 66, 67, 68, 69, 70).

Herein, we determined that baMntBC-A transports only manganese and not any other tested metals. Considering that baMntA is absolutely essential for the virulence of *B. anthracis* (10) means that the high-affinity acquisition of manganese is a major rate-limiting step in the progression of anthrax. This observation may have interventional potential as had been reported for PsaA of *S. pneumonia* (11). In this respect, it is noteworthy that the release kinetics of the metal from the SBP appear to play an essential role in determining the transport specificity on the one hand and the inhibitory potential on the other: for both *S. pneumonia* PsaA and baMntA, it was found that zinc is recognized with a similar affinity as manganese yet is released from the SBP at a much slower rate (24, 40). In both cases, zinc is not transported and acts as an inhibitor. This strategy of using slow-releasing cognate binders of SBPs as inhibitors may be generally applicable also to inhibition of other ABC transporters.

Nickel and cobalt are also recognized by baMntA, the latter with ∼10-fold higher affinity than zinc or manganese. Despite that, neither nickel nor cobalt inhibits manganese import by baMntBC. This difference likely stems from the preferred coordination number of the metals, as has been elegantly demonstrated for the PsaA of *S. pneumonia* (40). Manganese, cobalt, and nickel all prefer hexa-coordinate ligation, whereas zinc’s preferred coordination number is 4 (71, 72, 73). The tetrahedral coordination provided by the metal-ligating residues of baMntA optimally satisfies zinc’s preferred coordination, whereas binding of manganese, nickel, and cobalt is less favorable. This leads to an almost irreversible binding of zinc, yet reversible binding of manganese, nickel, and cobalt. This is perhaps also the reason why throughout evolution, amoeba, protozoa, and macrophages use zinc to kill phagocytosed bacteria rather than cobalt or nickel (74, 75). On the other side, bacteria use multiple approaches for evading zinc inhibition of manganese import. For example, MntR, the manganese homeostasis master regulator, responds only to manganese, and not to zinc (76). This means that MntA will not be unnecessarily exposed to zinc. In addition, bacteria do not live in a thermodynamic equilibrium reality but rather under constant pre-equilibrium conditions. In such a shifting reality, the faster *k*_*on*_ of manganese binding to MntA relative to zinc (24) may provide selectivity favoring the former.

The closest available structural templates for modeling of baMntBC were all type II ABC importers, suggesting that baMntBC adopts the type II fold. Similar conclusions can be drawn from the trRosetta template-independent modeling, which yielded a very similar structure. Additional observations also support the notion that ABC importers of transition metals are related to type II ABC importers. These include the similarity in their cognate SBPs structures, and the fact that unlike SBPs of type I systems, SBPs of transition metals do not undergo large conformational changes upon ligand binding (77, 78, 79). The high uncoupled ATPase activity of baMntBC-A (Fig. 6) also suggests a similarity to type II ABC importers (46, 57, 58). However, these results need to be interpreted with caution as this ATPase activity was measured in *E. coli* membranes that may lack unknown *B. subtilis* components.

In conclusion, our findings suggest that baMntBC-A and likely other ABC importers of transition metals contain unique motifs of two triads of titratable residues and a hydrophobic plug. We propose that these motifs have evolved specifically for the TM translocation of high-density charges. Considering their direct relevance to bacterial virulence and pathogenesis, and the emerging power of single-particle cryo-EM analysis, we propose that their structure–function analysis is timely.

## Experimental procedures

### Plasmids

The baMntBC-A operon was amplified by PCR from the genome of *B. anthracis* Vollum strain and cloned into the pDR111 (BGSC) vector for expression in *B. subtilis*, downstream to the IPTG-inducible promoter.

Point mutations were introduced into WT baMntBC-A by using the QuikChange Lightning Site-Directed Mutagenesis Kit (Agilent Technologies). Mutations were confirmed by sequencing.

### *B. subtilis* strains

**Table:** 

*B. subtilis*	Genotype and description	Origin
168	trpC2 (WT strain)	BGSC
*ΔmntR*	mntR::erm trpC2 (KO of strain 168 locus BSU24520)	BGSC

### Transformation to *B. subtilis*

To construct *B. subtilis* strains expressing baMntBC-A, a transformation protocol for genomic integration was used. Briefly, a single colony of WT or *ΔmntR B. subtilis* was picked from the LB plate into transformation buffer containing 1× MC buffer and 1 mM MgSO_4_ (10× MC buffer containing 0.62 M K_2_HPO_4_, 0.38 M KH_2_PO_4_, 20% (w/v) glucose, 30 mM trisodium citrate, 0.022 mg/ml ferric ammonium citrate, 1% (w/v) casein hydrolysate, and 2% (w/v) potassium glutamate) and incubated at 37 °C for 4.5 h with shaking. One microgram of DNA was added, and the sample was further incubated for 1.5 h at 37 °C. Samples were plated on LB plates supplemented with the 150 μg/ml spectinomycin, and plates were incubated at 37 °C overnight. Single colonies were picked, baMntBC-A insertion was verified by PCR, and positive colonies were kept in glycerol stock at −80 °C until use.

### Manganese sensitivity assays

*B. subtilis ΔmntR* transformed with control (pDR111) or baMntBC-A strains were grown in LB supplemented with 150 μg/ml spectinomycin at 37 °C. Cells were diluted to *A*_600_ of 0.05, and 0.15 ml cultures were grown with 1 mM IPTG in the absence or presence of the indicated metal in an automated plate reader (Infinite M200 Pro; Tecan). All metals were added as sulfate salts unless otherwise indicated. The absorbance of the cultures was measured every 10 min for 12 h. All assays were performed in triplicates.

For metal sensitivity assays on solid media, cells were grown overnight on LB supplemented with 150 μg/ml spectinomycin at 37 °C. Five dilutions were performed and applied dropwise (1.5 μl) on top of LB plates with the indicated metal concentrations.

### ICP-MS

Metals were added to midexponential phase cultures for 15 min at final concentrations of 50 to 1000 μM, as indicated (all metals were added as sulfate salts unless otherwise indicated). Cells were then harvested and washed with 1× PBS in the presence of 5 mM EDTA. The pellets were resuspended in 69% HNO_3_ and incubated in 100 °C dry block until complete evaporation. The remaining dry biomass was resuspended in 3% HNO_3_ to yield 5 to 250 ppm, and the final read was corrected for the dilution factor. All ICP-MS measurements were performed in biological triplicates using Agilent 7500cx ICP-MS with a dynamic range of 0.1 ppt to 2500 ppm.

### Preparation of inverted membrane vesicles

Cell pellets were washed once with 50 mM Tris HCl, pH 7.5, and 0.5 M NaCl and resuspended (20% w/v) in 50 mM Tris HCl, pH 7.5, 0.5 M NaCl, 30 μg/ml DNase (Worthington), one EDTA-free protease inhibitor cocktail tablet (Roche), 1 mM CaCl_2_, and 1 mM MgCl_2_ and ruptured first by tip-sonication and then using an EmulsiFlex-C3 homogenizer (Avestin) at 12,000 psi external pressure. Debris and unbroken cells were removed by centrifugation (10,000*g* for 20 min), and the membranes were collected by ultracentrifugation at 135,000*g* for 30 min, washed once in 25 mM Tris HCl, pH 7.5, 0.1 M NaCl, and resuspended in the same buffer to ∼1 mg/ml.

### ATPase assays

ATP hydrolysis was measured using Molecular Probes EnzChek kit, at 37 °C, in a 96-well format, according to the manufacturer’s specifications. To initiate hydrolysis, 5 mM MgCl_2_ was injected to a solution containing 15 μg of inverted membrane vesicles in 25 mM Tris HCl, pH 7.5, 0.1 M NaCl, 50 μM EDTA, and the indicated ATP concentration. Data were fitted using either the Michaelis–Menten equation or its expanded version, which includes also a term for the Hill coefficient:

$$V=V_{\max}\frac{{\left[S\right]}^{n}}{{\left[S\right]}^{n}+K_{m}}$$

Where *V* is the observed hydrolysis rate, *V*_max_ is the maximal hydrolysis rate, *K*_*m*_ is the Michaelis–Menten constant, [*S*] is the concentration of ATP, and *n* is the Hill coefficient.

### baMntBC modeling

#### Searching for structural templates

The amino acid sequences of baMntB and baMntC were used as queries in two independent HHpred (80) searches. For baMntB, the best scoring templates included the NBDs of *H. influenza’s* putative molybdate/tungstate importer MolBC in an inward-facing conformation (PDB ID 2NQ2); *Y. pestis’s* heme importer HmuUV in an outward-apo conformation (PDB ID: 4G1U); and *E. coli’s* vitamin B_12_ importer BtuCD (PDB ID: 4R9U). All three templates showed a 28% sequence identity to baMntB. The best scoring templates for baMntC included the same three templates collected for baMntB, only with much lower sequence identity, specifically 14%, 15%, and 13% for 2NQ2, 4G1U, and 4R9U, respectively.

#### Sequence search and multiple sequence alignment

ConSurf (50) was used to collect independent sets of homolog sequences for baMntB and baMntC and each of the two domains (NBD and TMD) of the three structural templates. Homolog searching was conducted using HMMER (81), with an E-value of 0.0001, maximal sequence identity of 95%, and minimal sequence identity of 15% against the clean UniProt database. 500 sequences that sample the list of homologs were chosen automatically by ConSurf and were aligned using MAFFT (82), with default parameters. Fragmented sequences and sequences that introduced large gaps in the multiple sequence alignment (MSA) were then manually removed, and the remaining sequences were realigned using MAFFT. The final MSAs contained 410 homologs for baMntB; 430 homologs for baMntC; 374 homologs for 2NQ2’s NBD and 434 for its TMD; 355 homologs for 4G1U’s NBD and 355 for its TMD; and 370 homologs for 4R9U’s NBD and 392 for its TMD.

Next, MAFFT was used to perform profile-to-profile MSA between baMntB/baMntC and each of their three templates. From these MSAs, pairwise alignments between baMntB/baMntC and each template were deduced.

#### Improving the pairwise alignment between baMntC and its templates

Given the low query-to-template sequence identity between baMntC and its templates, we used evolutionary conservation analysis alongside multiple computational tools to independently assign TM helices and manually improve our pairwise alignments. Specifically, we used HMMTOP, Phobius, TMHMM, TopGraph (83), MEMSAT-SVM (84), TOPCONS (85), RaptorX (86, 87), and ConSurf (50) (Figs. S4 and S6). Adjustments were made mainly in the regions of the second and fourth predicted helices (amino acids 40-to-67 and 89-to-115, respectively). The improved pairwise alignments were then used for the modeling process.

#### Constructing the 3D models

MODELLER 9.18 (88) with default settings was used to produce the 3D models of baMntB. Each model was built using one template. For constructing the 3D models of baMntC, each of the three templates identified by HHpred was used alongside an *ab initio* model produced by RaptorX. The latter was mainly used to model gapped regions such as the N and C termini. For each model, 100 different structures were produced. A short energy minimization was conducted using GROMACS 5.1 (89) and the AMBER99SB-ILDN force field (90). The model with the predicted lowest energy was then selected.

An additional template-independent model was constructed using trRosetta (48) that models a protein’s structure using neural network to predict inter-residue distance and orientation distributions based on coevolution data. Default parameters were used with the no-template option. The resulting model was subjected to a short energy minimization similar to the homology models. Overall, both trRosetta and the homology modeling converged to similar structures. The main difference between the models was the position of TM-4 relative to the other helices (Fig. S5*A*). As such, two (F105 and I109) of the four residues suggested to compose MntC’s hydrophobic ladder face the core of each monomer rather than the interface (Fig. S5*B*). The overall RMSD when superimposing trRosetta’s model to the three homology models ranged between 4.83 and 5.6 Å (Table S1). Importantly, the trRosetta calculations account for evolutionary coupling only within the chain. Thus, coupling between the two monomers in MntC’s structure is not taken into account. This means that any constrains that relate to the translocation pathway situated in the interface between the two monomers are ignored by the trRosetta calculations.

### **Note added in proof**

After this article was accepted for publication, the X-ray crystal structure of a close homolog of baMntBC was published, namely, *Streptococcus pneumoniae* PsaBC manganese transporter (PDB entry: 7KYP; PMID: 34362732). Notably, the three baMntBC models presented in this work, which were constructed based on remote homologs, show remarkable resemblance to this new structure. Specifically, the membrane domains superimpose with an RMSD of 3.4 to 3.8 Å and the ATPase domains with an RMSD of 1.8 to 2.0 Å. Importantly, the orientation of residues consisting the hydrophobic ladder and those proposed to mediate ion coordination was predicted with high accuracy. Thus, the *Streptococcus pneumoniae* PsaBC structure further consolidates the hypotheses and conclusions presented in this work.

## Data availability

The data that support the findings of this study are available from the corresponding author upon reasonable request.

## Supporting information

This article contains supporting information

## Conflict of interest

The authors declare that they have no conflicts of interest with the contents of this article.
