# Supplementary material for: Titratable transmembrane residues and a hydrophobic plug are essential for manganese import via the Bacillus anthracis ABC transporter MntBC-A
Source: J Biol Chem. 2021 Aug 18;297(4):101087. doi: 10.1016/j.jbc.2021.101087 (PMC8487065; doi:10.1016/j.jbc.2021.101087)
Supplement: Supplemental Figures S1–S7 and Table S1 [file mmc1.pdf]

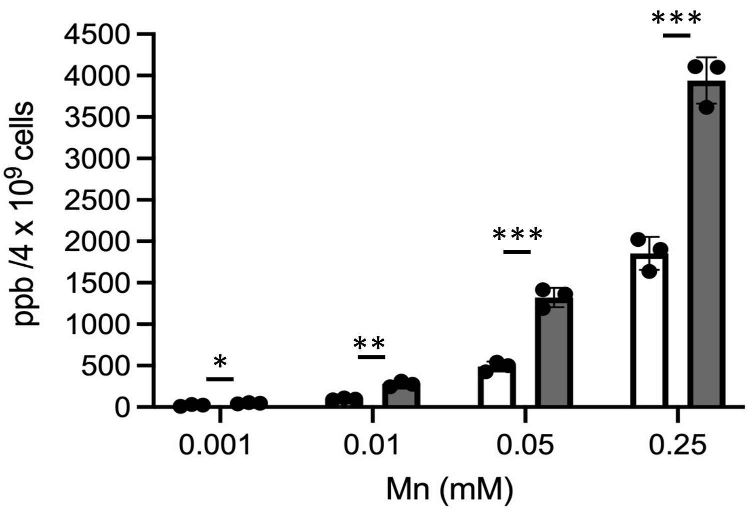

Supplementary Figure 1. Metal accumulation mediated by baMntBCA. Mid exponential phase cultures of *Bacillus subtilis* cells transformed with either a control plasmid (open bars) or a plasmid harboring the complete baMntBCA operon (grey bars) were incubated for 15 min with the indicated concentrations of $MnSO_4$. Cells were then harvested, washed with PBS-EDTA buffer and their intracellular Mn^{2+} content was determined by ICP-MS. Shown are averages of technical triplicates of experiments conducted at least three times. Error bars represent the standard deviations. * $p<0.05$; ** $p<0.01$; *** $p<0.001$;two-tailed student t-test.

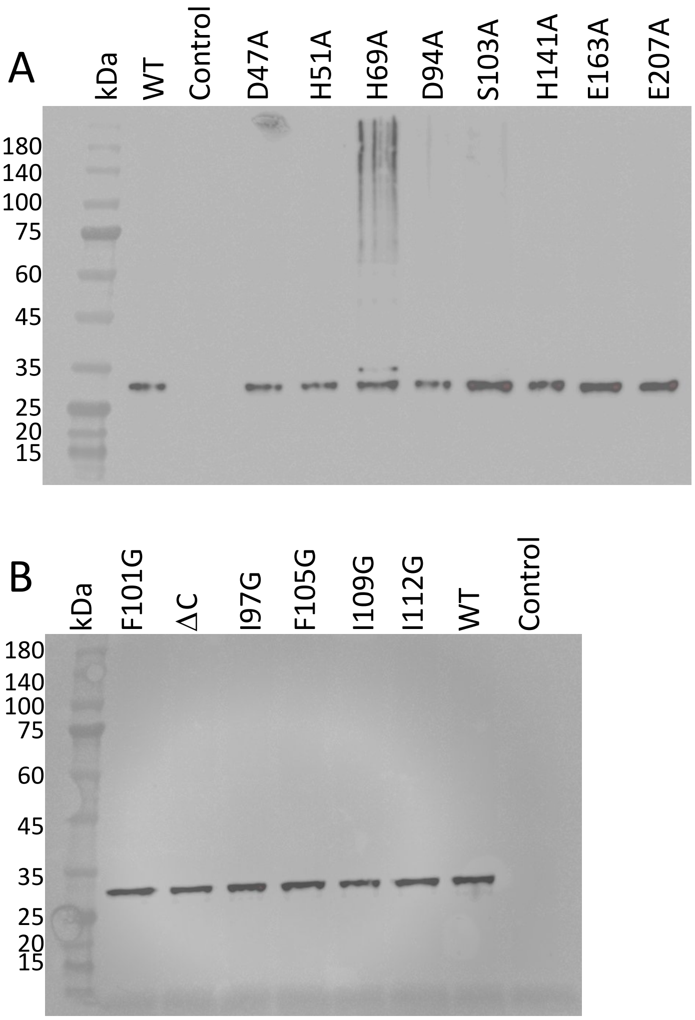

Supplementary Figure 2. Expression of WT and mutant baMntBC-A. Membranes fractions were prepared from cells transformed with an empty integration vector (control), or a plasmid encoding WT $_{BA}$mntBCA or the indicated mutants. 1 µg of total membrane protein were separated by SDS-PAGE and the protein content was visualized by immuno-blotting using an a-His antibody. (A) Expression of mutants of the metal binding residues of MntA and titratable residues of MntB. (B) Expression of mutants of the hydrophobic seal and the C-terminal truncation (ΔC, as indicated).

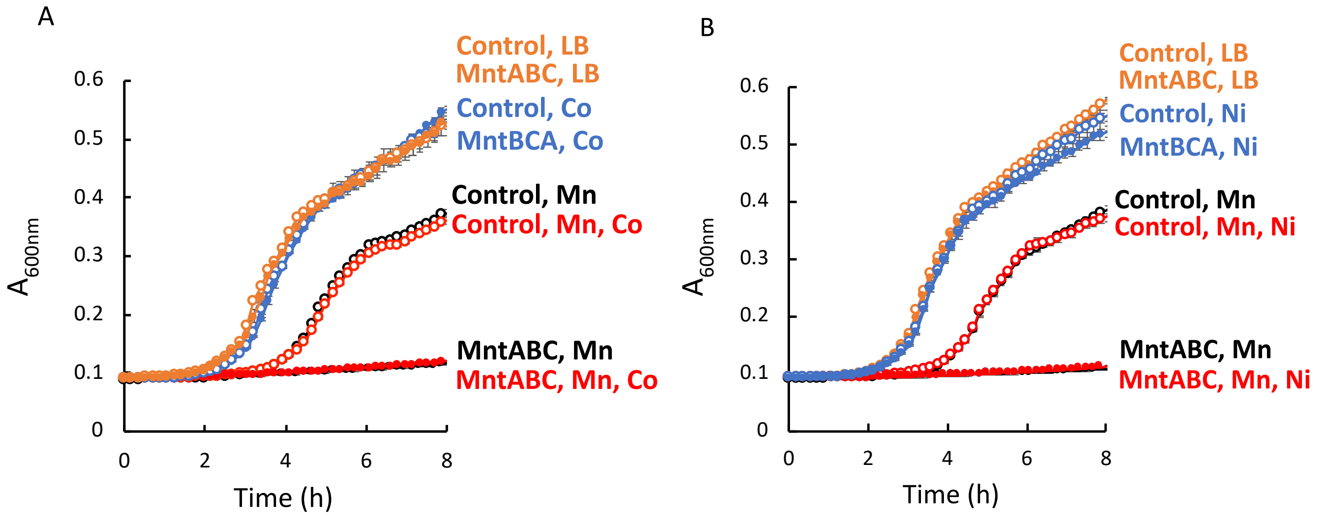

Supplementary Figure 3. Cobalt and nickel do not inhibit baMntBC-A-mediated transport of manganese. (A) Cultures of *Bacillus subtilis ΔmntR* cells transformed with a control plasmid (open symbols) or a plasmid harboring the complete baMntBC-A operon (full symbols) were grown in LB media (orange symbols) or LB media supplemented with 10 μM $MnSO_4$ (black symbols), 50 μM $CoSO_4$ (blue symbols), or their combination (red symbols). (B) Same as 'A', only with $NiSO_4$. Shown are averages of biological triplicates, error bars (shown unless smaller than the symbols) represent standard deviations of the mean.

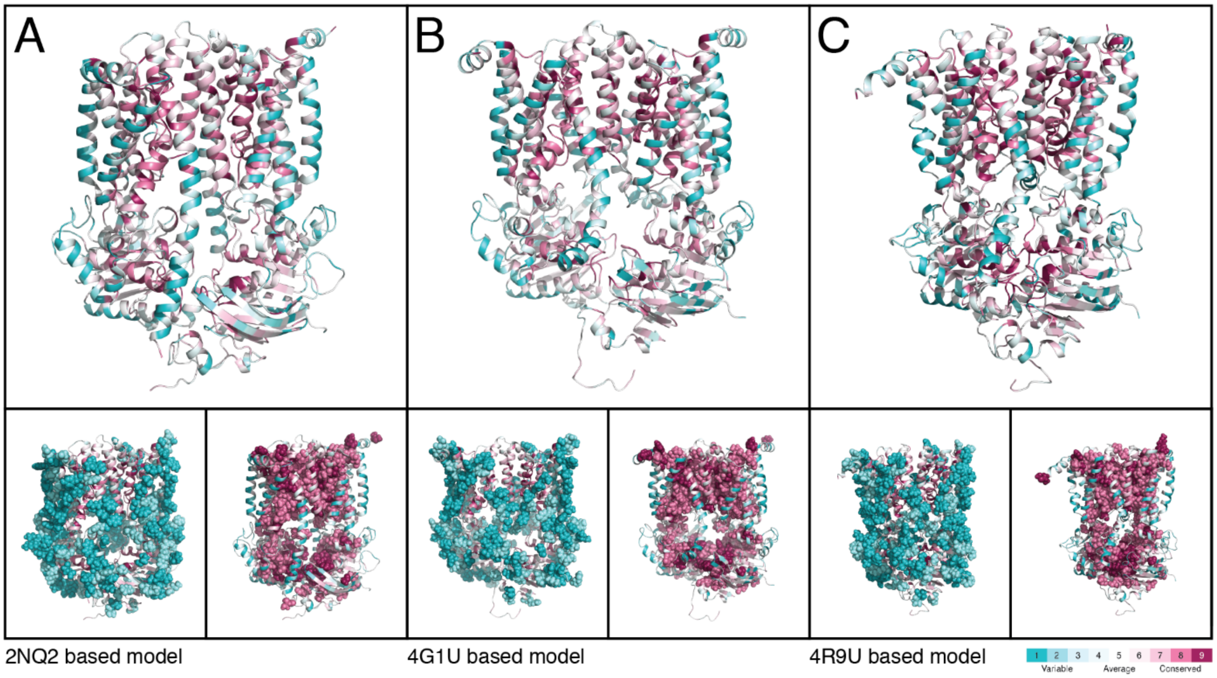

Supplementary Figure 4. Homology models of baMntBC and their evolutionary conservation. 3-D models of baMntBC were generated by homology modelling using three different templates (PDB IDs 2NQ2 (A), 4G1U (B) and 4R9U (C)). The models are shown as a cartoon representation (upper panel) and colored according to evolutionary conservation. Cyan represents the most variable positions and maroon the most conserved. The lower panel shows the distribution of the most variable positions compared to the most conserved ones, for each model, in spheres representation. Reassuringly, the models are consistent with the expected evolutionary conservation pattern, where the protein's core is composed almost exclusively of conserved residues, while its periphery is enriched with variable residues.

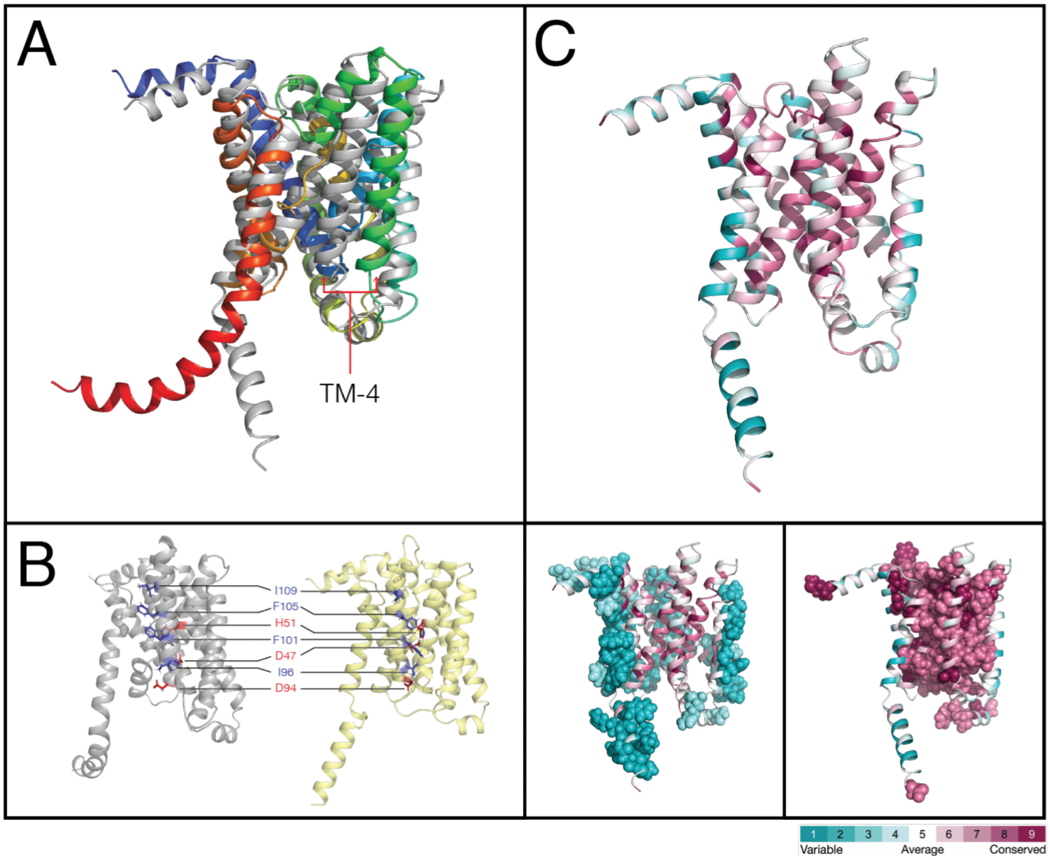

Supplementary Figure 5. Template-independent modelling of baMntBC. Template independent model of (monomeric) baMntC produced using trRosetta. (A) Cartoon representation of trRosetta's model (in rainbow representation) superimposed to the 4G1U-based homology model (in gray). The RMSD between the two models is 4.59 angstrom, with the main difference being the position of TM-4 relative to the other helices. (B) Cartoon representation of trRosetta's model (yellow) and the 4G1U-based homology model (gray) with the charged triad and the hydrophobic ladder shown in sticks representation and colored red and blue respectively. (C) Evolutionary conservation pattern of trRosetta's baMntC model shown in cartoon representation (upper panel) and spheres representation (lower panel) colored according to evolutionary conservation as in Supplementary Figure 4.

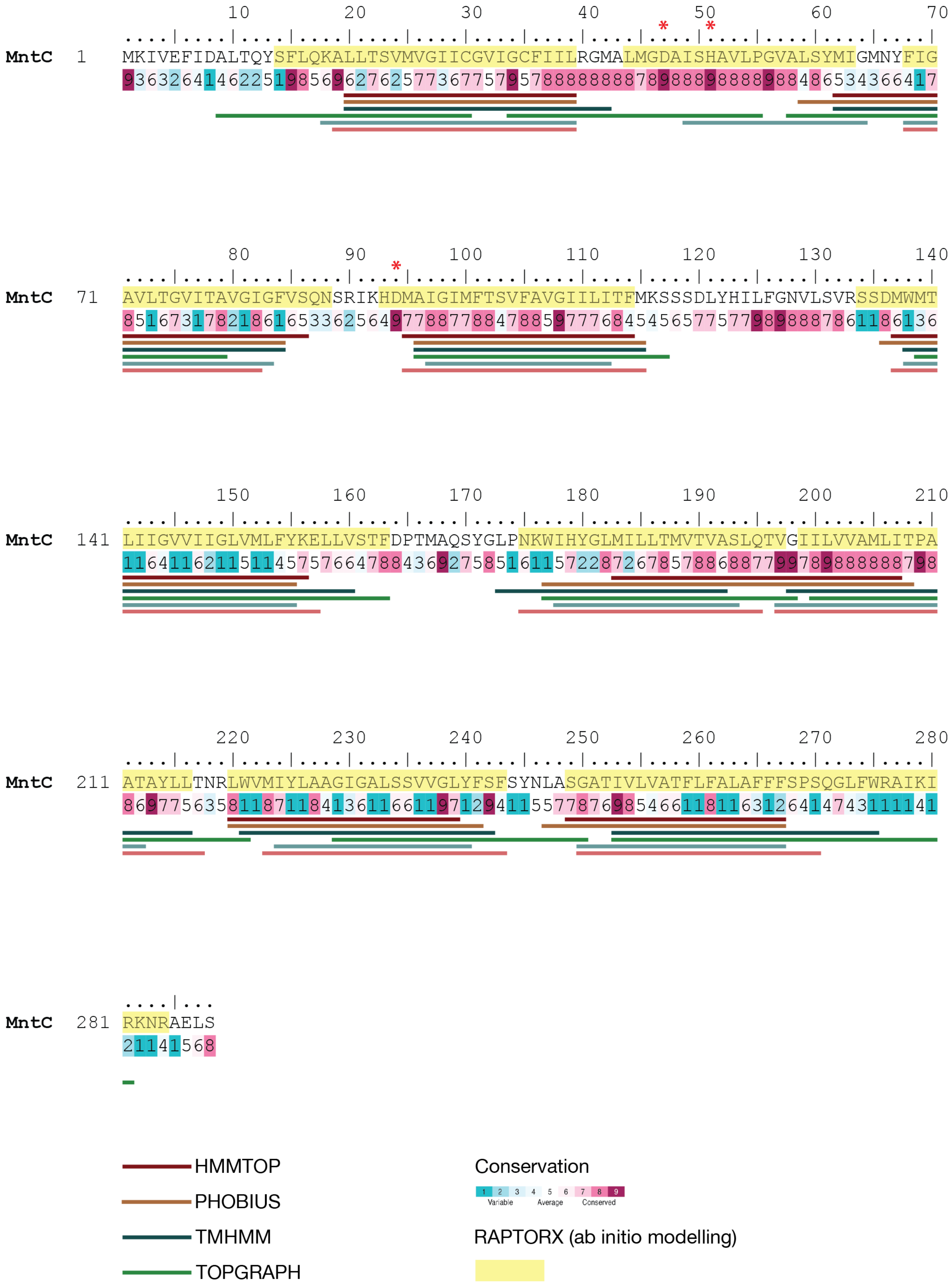
10 20 30 40 50 60 70
MntC 1 MKIVEFIDALTQYSFLQKALLTSVMVGIICGVIGCFIILRGMALMGDAISHAVLPGVALSYMIGMNYFIG
9363264146225198569627625773677579578888888878988898888988486534366417
80 90 100 110 120 130 140
MntC 71 AVLTGVITAVGIGFVSQNSRIKHDMAIGIMFTSVFAVGIILITFMKSSSDLYHILFGNVLSVRSSDMWMT
8516731782186165336256497788778847885977768454565775779898887861186136
150 160 170 180 190 200 210
MntC 141 LIIGVVIIGLVMLFYKELLVSTFDPTMAQSYGLPNKWIHYGLMILLTMVTVASLQTVGIILVVAMLITPA
1164116211511457576647884369275851611572287267857886887799789888888798
220 230 240 250 260 270 280
MntC 211 ATAYLLTNRLWVMIYLAAGIGALSSVVGLYFSFSYNLASGATIVLVATFLFALAFFFSPSQGLFWRAIKI
8697756358118711841361166119712941155778769854661181163126414743111141
MntC 281 RKNRAELS
21141568
HMMTOP
PHOBIUS
TMHMM
TOPGRAPH
MEMSAT-SVM
TOPCONS
Conservation
1 2 3 4 5 6 7 8 9
Variable Average Conserved
RAPTORX (ab initio modelling)

Supplementary Figure 6. Conserved residues of baMntBC and assignment of transmembrane helices for baMntC. The amino acids sequence of baMntC annotated with the putative transmembrane helices predicted by different computational tools. Namely, HMMTOP, PHOBIUS, TMHMM, TOPGRAPH, MEMSAT-SVM, TOPCONS and RAPTORX. For each amino acid in the sequence, its conservation grade, based on ConSurf calculations, is shown as numbers (1-to-9) with corresponding colors (cyan-through-maroon). Cyan (1) represents the most variable positions and maroon (9) the most conserved. The titratable residues that are suggested by the models to line the translocation cavity (D47, H51 and D94) are marked with red asterisks.

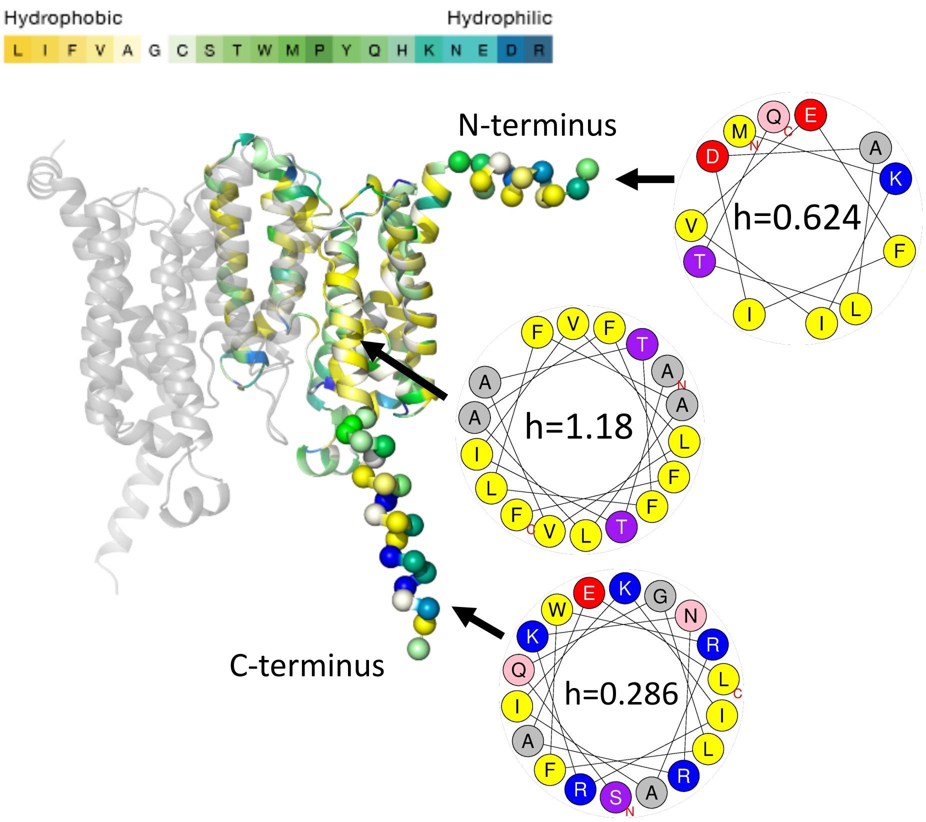

Supplementary Figure 7. Hydrophobicity analysis of baMntC. (A) baMntC model structure in cartoon representation with one monomer colored according to Kesesl & Ben-Tal's hydrophobicity scale. The N- and C-termini are shown in spheres representation. **(B)** Helical wheel representation and Roseman hydrophobicity scale of the N- and C-termini and of a representative TM helix (helix 9), as indicated. As shown, the N-terminus helix is amphipathic with intermediate hydrophobicity, the TM helix is highly hydrophobic and not amphipathic, and the C-terminus is highly hydrophilic and not amphipathic.

Supplementary Table 1. RMSD between the four baMntBC models.

	trRosetta's model	2NQ2 based model	4G1U based model
2NQ2 based model	5.6 Å		
4G1U based model	4.83 Å	3.5 Å	
4R9U based model	5.48 Å	2.9 Å	4.36 Å
